# Assessing the mental health impacts of Israeli occupation infrastructure in the West Bank by combining geospatial data with a representative survey of Palestinian youth

**DOI:** 10.1016/j.healthplace.2025.103420

**Published:** 2025-02-22

**Authors:** Nadia Almasalkhi, Peter Glick, Samer Atshan, Wenjing Huang, Jad Isaac, Umaiyeh Khammash, Daniel Egel

**Affiliations:** a RAND Corporation, 1200 S Hayes St, Arlington, VA, 22202, USA; b Pardee RAND Graduate School, 1776 Main Street, Santa Monica, CA, 90401, USA; c RAND Corporation, 1776 Main Street, Santa Monica, CA, 90401, USA; d Applied Research Institute – Jerusalem, P.O. Box 860, Karm Mu’ammar, Karkafeh Street, Bethlehem, Palestine; e Juzoor for Health and Social Development, Al-Arkan St, Al-Bireh, West Bank, Palestine

**Keywords:** Mental health, Youth, Occupied Palestinian Territory, Behavioral health, Built environment

## Abstract

Palestinian youth in the West Bank, occupied Palestinian territory (oPt) live in proximity to various forms of Israeli occupation infrastructure, such as checkpoints, road obstructions, a separation barrier, and Israeli settlements. We investigated the effect of proximity to such infrastructure on youth mental health and health risk behaviors by linking geospatial data on the locations of occupation infrastructure to geocoded survey data collected from a representative sample of Palestinian youth living in the West Bank. We estimated the relationship of youth mental health and proximity to each type of occupation infrastructure with controls for exposure to conflict-related violence and a range of demographic factors. We found that youth mental health is strongly negatively impacted by proximity to manned checkpoints (for males and females) and proximity to settlements (for females), and these impacts appear to be direct rather than mediated by conflict-related trauma exposure. The results indicate the importance of environmental aspects of the conflict for youth mental health, in addition to conflict-related violence itself.

## 1. Introduction

Political conflicts have long-term and debilitating effects on the mental health of more than 140 million children living in conflict across the globe (The Lancet Editorial Team, 2019). These populations suffer from extremely high rates of mental health disorders, which can contribute to substance abuse, violent behavior, worsened physical health, and reduced psychological functioning (Murthy and Lakshminarayana, 2006; Neuner and Elbert, 2007). A wide range of studies have demonstrated that the Israeli-Palestinian conflict and the context of Israeli occupation of the West Bank contributes to psychological risk for Palestinian children and youth. Most of this research has studied the effects of direct exposure of young people to conflict-related trauma and physical violence (Abdeen et al., 2008; Al-Krenawi et al., 2007; Ayer et al., 2017; Khamis, 2005, 2012; Sagi-Schwartz, 2008; Veronese et al., 2022, 2023; Wagner et al., 2020).

However, conflict may affect health and well-being not just through direct exposure to violent conflict or conflict-related trauma (e.g., one-self or a family member being arrested, injured, or tortured), but also through broader aspects of the conflict environment. Studies in diverse conflict settings show that a significant share of the sample variance in mental health outcomes remains unexplained after accounting for direct war exposure, and that accounting for ‘daily stressors’—that is, “the stressful social and material conditions that are often caused or exacerbated by armed conflict” (Miller and Rasmussen, 2010a, p. 1385)—helps explain that variance (Miller and Rasmussen, 2010b). This suggests the need for ecological approaches, which consider multiple chronic influences on mental and behavioral health at the individual, household, and community levels. Using this broader lens, potential indirect pathways through which conflict environments may impact wellbeing include increased economic hardship; constraints on mobility and livelihoods; reduced access to health services and lessened availability of other goods and services; lawlessness and insecurity; and breakdown of community cohesion and support systems (Fernando et al., 2010; Miller, 1999; Miller et al., 2008; Rasmussen et al., 2010). The conditions created by political conflict thus introduce a variety of daily stressors that can negatively affect young people’s mental health. Poor mental health and pessimism about the future in turn may further endanger young people’s wellbeing by encouraging engagement in drug use and other risk behaviors, whether as a coping mechanism or as a consequence of feeling that there is “nothing to lose” (Garland et al., 2013; Harris et al., 2002; Leeies et al., 2010).

The present study adopts an ecological approach to understanding the impact of conflict on youth wellbeing. Specifically, we consider how Israeli occupation infrastructures, as chronic features of the conflict setting, may affect Palestinian youth mental health and risk behaviors separately from exposure to conflict-related violence. This system of infrastructure comprises, at the time of our data collection, (1) a network of 77 checkpoints manned by Israeli security forces, including both government forces and private security companies (Kennard, 2016); (2) 310 unmanned obstacles that obstruct the internal movement of Palestinians; (3) a separation barrier designed to isolate West Bank Palestinians from Israel, which consists of 70 km of 8–9 m high concrete walls (in urban areas) and an additional 395 km of an approximate 60-m wide land barrier with barbed wire electric fences on both sides; and (4) a constellation of 132 Israeli settlements that are legal under Israeli law and an additional 140 settlements that are illegal under Israeli law, through which Israeli settlers have come to constitute approximately 13% of the West Bank population by 2014 (Palestinian Central Bureau of Statistics, 2014; Peace Now, n.d.).^1^

We examine the role of conflict-related infrastructure specifically because of its wide-reaching effects on the daily lives of Palestinians, which may contribute to stressful daily living conditions. Previous studies and reports have suggested that mobility-restricting infrastructure like checkpoints and road obstacles have adverse effects on physical health, financial security, and child and youth development, as they can impede or delay access to medical facilities (High Commissioner for Human Rights, 2005), limit access to the labor market (Calì and Miaari, 2018), and cause young people to lose instructional hours in school (Global Coalition to Protect Education from Attack, 2022; UNICEF State of Palestine, 2018; United Nations Office for the Coordination of Humanitarian Affairs - oPt, 2018). Further, a much noted aspect of life under the occupation is that encounters with Israeli forces at checkpoints often involve harassment and humiliation (Barber et al., 2016; Giacaman et al., 2007), and potentially, beatings and tear-gassings (Karakashian, 2019; United Nations Office for the Coordination of Humanitarian Affairs - oPt, 2020, 2019). With regard to settlements, the literature has also shown that the construction of settlements in the West Bank is associated with property loss, poverty, reduced clean water access, and added mobility restrictions for Palestinians, which are correlated with negative physical health outcomes in children (Fahoum and Abuelaish, 2019). Additionally, Palestinians living in close proximity to Israeli settlements may experience harassment, property destruction, home invasions, and threat of forced expulsion (Sousa et al., 2014; United Nations Office for the Coordination of Humanitarian Affairs - oPt, 2020). Finally, with regard to the separation barrier, previous research on the West Bank and on Northern Ireland provides reasons to suspect that separation barriers and related infrastructure may impact mental health by heightening perceptions of social exclusion (Maguire et al., 2016) and exacerbating poverty (Sadeq and Lubrano, 2018).

This study conceptualizes Israeli occupation infrastructure as a potential source of daily stressors for youth, which can have impacts on their mental health and health risk behaviors. We take a novel approach to studying this relationship, using distance to such infrastructure to capture the intensity of youth’s exposure to these stressors. Very few prior studies on the oPt have used spatial data to directly model the effects of proximity to checkpoints, barriers, and settlements; those that have done so focus on economic and labor market impacts (Calì and Miaari, 2018; Miaari and Milosav, 2023; Sadeq and Lubrano, 2018; van der Weide et al., 2018). Despite not using spatial data, recent studies on how the occupation impacts mental health in the West Bank nevertheless point to the importance of mobility and spatial arrangements (Cavazzoni et al., 2022; Giacaman et al., 2023; Hammoudeh et al., 2022; Veronese et al., 2020). Notably, in their analysis of survey data, Hammoudeh et al. (2022) use multi-level modeling to distinguish between mental health effects produced at the individual-, household-, or locality-level, and find that locality accounts for 11.4% of the variance in adults’ mental health outcomes. However, their analysis does not include measures of locality-level variables and thus cannot identify the specific locality-level factors driving the significant variation they find across space (Hammoudeh et al., 2022). Our study addresses their call to investigate how spatial characteristics in the West Bank influence mental health (Hammoudeh et al., 2022) by estimating how proximity to checkpoints, road obstructions, the separation barrier, and settlements—features that are unevenly distributed across space—are correlated with youth mental health and risk behaviors.

The present study contributes a unique spatial analysis of the occupation environment’s effects on mental and behavioral health by combining geocoded data from a large-scale representative survey of youth ages 15–24 in the West Bank with spatial data on checkpoints, road obstructions, the separation barrier, and settlements. We use these data to assess the occupation environment’s effects on depression and anxiety, smoking, pre-marital sex, alcohol use, and illicit drug use. Because the youth survey collected detailed information on respondents’ individual and family experiences of conflict-related trauma, we are able to estimate the independent effects of the occupation environment, controlling for conflict-related violence exposure. By examining the effects of proximity to occupation infrastructure alongside the effects of conflict-related trauma, this article builds on prior large-*N* representative studies of youth mental health in the West Bank that have primarily focused on the latter (Abdeen et al., 2008; Al-Krenawi et al., 2007; Khamis, 2005, 2012; Wagner et al., 2020). This study thus aims to fill a critical gap in the existing literature by examining how barriers to mobility and other chronic features of the Israeli occupation affect the mental and behavioral health of Palestinian youth. This research seeks to quantify these relationships and thus contribute to a layered understanding of how the Israeli-Palestinian conflict in the West Bank shapes the psychological well-being and behavior of young Palestinians. Ultimately, our findings aim to inform policymakers, scholars, and practitioners on the multifaceted effects of low-intensity, protracted political conflicts on youth mental health, highlighting the need for targeted interventions that address both their direct and indirect impacts.

## 2. Methods

### 2.1. Survey data and procedures

Our study links geospatial data on Israeli infrastructure to individual-level data from the Palestinian Youth Health Risk Survey, a representative survey that provides information on self-reported conflict-related trauma, mental health, and health risk behaviors in addition to standard demographic information such as education, employment, and household assets. The data comprise a sample of 2229 youth aged 15–24 years in the West Bank (excluding East Jerusalem) surveyed between April and July 2014 (Glick et al., 2018). The survey was stratified geographically by governorate and urban, rural, and refugee camp locality type. Within each geographic stratum, census enumeration areas were randomly sampled, with the probability of selection proportional to population size. Across the 11 governorates of the West Bank, 187 census enumeration areas were selected as survey clusters.^2^ Within each cluster, survey interviewers selected 12 households with youth via the random walk method, using implicit stratification to ensure equal numbers by gender.^3^ In each household, one youth was interviewed; in households where there was more than one youth between 15 and 24 years old, one was selected at random using a Kish selection table. Parental verbal consent was obtained to interview minors, in addition to obtaining verbal consent to participate from all youth. Verbal consent was obtained instead of written consent in order to protect participants’ confidentiality. The refusal rate was 8%. Surveys were administered face-to-face by an interviewer of the same gender as the respondent in a private location (e.g., in a private room of the house, on the roof; youth were also given the option to have the interview at a separate location outside of the home). To ensure confidentiality, no respondent or family names or addresses appeared on any questionnaire forms used by the survey.

Survey teams collected latitude and longitude data for the approximate centroid of each cluster. (See Appendix Section A2 for further details on survey methods). These geographic data allow us to generate a cluster-specific measure of proximity to each of the four types of occupation-related infrastructure included in our analysis.

### 2.2. Infrastructure data

This study uses geospatial data from multiple sources to map the locations of checkpoints, unmanned road obstructions, the separation barrier, and Israeli settlements as of 2014, to align with the timing of the youth survey. The locations of checkpoints and road obstacles were mapped using a geospatial historical database developed by the Applied Research Institute of Jerusalem. Spatial data on the separation barrier came from United Nations Office for Coordination of Humanitarian Affairs. Data on the locations of Israeli settlements were provided by B’Tselem, an Israeli human rights organization.

### 2.3. Independent variables

Our primary measures of proximity are the natural logarithms of the Euclidean (straight line) distance from each survey cluster centroid to the closest checkpoint, unmanned obstruction, segment of the separation barrier, and settlement. As proximity is measured at the level of the survey cluster, it can be considered a community-level variable, in line with multi-level ecological models. Given the novel nature of an analysis using these variables, and the wide range of approaches used in public health research to measure geographic proximity (Chakraborty et al., 2011), we conduct sensitivity analysis using alternative measures of distance to these physical infrastructure types. Our sensitivity analysis considers the following alternatives: a distance-weighted index used by Calì and Miaari (2018) in their study of mobility restrictions in the West Bank; a dichotomous approach as used by Maguire and others in studying the physical manifestations of segregation in Northern Ireland that focuses on circular buffers zones of various sizes (Maguire et al., 2016); the quantity of infrastructure pieces within a given radius; and different continuous specifications including linear Euclidean distance and inverse Euclidean distance. (See Appendix Section A1 for definitions and details).

### 2.4. Outcome variables

Our primary outcome variable was youth mental health. The youth survey implemented the Hopkins Symptom Checklist-25 (HSCL-25), which has been validated as an interviewer-administered tool for detecting mental health disorders in many contexts, including the West Bank (Afana et al., 2002). The HSCL-25 contains two subscales: a 10-item scale focused on symptoms of anxiety and a 15-item scale focused on symptoms of depression, with the responses for each item captured in a 4-point Likert format. The 25 items together constitute an overall measure of global mental health (Ventevogel et al., 2007). While we focus primarily on global mental health, which aggregates the measures of anxiety and depression, we also examined the separate effects on depression and anxiety. For each measure, we summed across all items and then divide by the number of answered items to derive the index.

Our secondary outcomes were risk behaviors, for which data was also collected in the youth survey. The measures are dichotomous indicators for several forms of substance use (ever use illicit drugs, ever drink alcohol, and current tobacco smoking using either cigarettes or water pipe) and for engagement in pre-marital sexual intercourse (referred to below as sexual intercourse, for brevity). Questions about sexual intercourse were asked only to respondents aged 18 years or older. For more details on these measures, see Glick et al. (2018). Pairwise correlations between all outcome variables are shown in Appendix Table A1.

### 2.5. Covariates

In line with previous work on the effects of violence exposure on mental health, we adjusted for individual-level demographic and socioeconomic characteristics (Wagner et al., 2020). Demographic variables included age, gender, marital status, location of residence (binary indicators for refugee camp and urban setting, with rural as reference category), and separate variables for whether an individual’s mother or father are deceased. Socioeconomic characteristics include an estimate of household assets (Glick et al., 2018), the youth’s years of education, and binary indicators for current school enrollment, employment status, and whether the individual’s father, if living, was unemployed, disabled, or ill at the time of the interview.

As our focus is on identifying the impact of the conflict environment beyond an individual’s personal experience with conflict-related trauma, we also controlled for individual exposure to trauma. Following the existing literature, we divide experienced trauma into direct and vicarious exposure (Stein et al., 2003). We measured direct exposure to conflict-related trauma as the unweighted average of four binary variables indicating whether an individual had ever been: physically assaulted by soldiers or the police; shot by plastic, rubber, or real bullets; been imprisoned; or had a home closed or demolished by Israeli authorities. Vicarious exposure was measured as the unweighted average of three binary variables indicating whether an individual had ever: witnessed the shooting of a friend or relative; had a close relative imprisoned; or directly witnessed the demolition of a friend or relative’s home.

### 2.6. Statistical analysis

We first estimated unadjusted associations between the outcomes and proximity to the physical infrastructure measures using bivariate regression models. We then estimate a multiple regression model of the following form:

$$Y_{is}=\alpha_{0}+\alpha_{1}+g\left(\text{Physical}\;\text{Infrastructur}e_{s}\right)+\beta X_{is}+\varepsilon_{is}$$

Where *i* indexes individuals and *s* indexes survey clusters. *Y*_*is*_ refers to the outcome variable, *g*(*Physical Infrastructure*_*s*_) refers to the functional form that we use for estimating the proximity of each survey cluster to the different types of physical infrastructure, and *X*_*is*_ is comprised of the individual-level covariates described previously. We clustered all standard errors at the level of the survey cluster. For mental health, *Y*_*is*_ is a continuous measure, and we conduct ordinary least squares (OLS) regressions. For risk behavior measures, *Y*_*is*_ are binary indicators, so we estimate probit regressions and calculate the effects of a unit change in proximity on the probability that *Y*_*is*_ = 1 (i.e., marginal effects) when all other variables are set to their means.

We conducted sensitivity analyses using alternative specifications of the functional form used for measuring proximity for each infrastructure type, given that there is no standardized approach for measuring distance in this context, as noted above. Our primary measure uses the natural logarithm of the Euclidean (straight-line) distance from the centroid of the survey cluster to the closest checkpoint, road obstruction, segment of the separation barrier, or settlement. Our sensitivity analysis considered the following alternatives (details in Appendix Section A1): Euclidean distance; inverse distance; dichotomous variables indicating the presence (or not) of a checkpoint, obstruction, wall segment, or settlement within 1- or 2-km; the total number of checkpoints, obstructions, or settlements within 5-, 10-, or 20-km; and the sum of the inverse distance of infrastructure elements within 5-, 10, or 20-km.

### 2.7. Role of the funding source

The funders of the study had no role in study design, data collection, data analysis, data interpretation, or writing of the study. The corresponding author had full access to the data in the study and had final responsibility for the decision to submit for publication.

## 3. Results

### 3.1. Descriptive statistics

West Bank Palestinians on average live in close proximity to all four types of physical infrastructure of the occupation. For the 2229 youth included in the 187 survey clusters in the sample, the mean distance to the closest checkpoint manned by Israeli security forces is 4.1 km (range 0.5–12.6 km), to the closest Israeli-constructed physical obstruction blocking a road is 3.7 km (range 0.4–17.5 km), to the closest point on the separation barrier is 6.9 km (range 0.05–24.0 km), and to the closest Israeli settlement is 3.9 km (range 0.5–16.7 km). The cutoff points for each decile of distance to each type of infrastructure are presented in Appendix Table A2; the following sections will sometimes refer to ‘interdecile’ comparisons, which represent the difference between the tenth percentile and the ninetieth percentile of distance to a given type of infrastructure.

Table 1 shows descriptive summary statistics for the full sample and separately for the female and male subsamples. Notable differences by gender are seen for educational attainment, employment, marital status, assets, and trauma exposure. Female youth tended to have higher educational attainment than male youth, and were more likely to be still enrolled in school, while male youth were more likely to be employed. Females in this sample were much more likely than males to be married; this may reflect a trend in which married female youth have somewhat older spouses who are more likely to be beyond the upper age limit of the sample. The mean asset index among male youth is markedly higher than among female youth, which may relate to this marriage differential: more females in the sample are married and have formed new households which have not accumulated much wealth, while more males in the sample remain part of their parents’ household. Notably, self-reports of both direct and vicarious trauma exposure were higher among males than females.

Adverse mental health outcomes are prevalent among the youth in this sample. Previous research with Palestinians has established that a HSCL-25 score of 1.75 or greater is indicative of a mental health disorder (Afana et al., 2002); the mean HSCL-25 for males in our survey was 1.67 (SD 0.40) and for females was 1.93 (SD 0.50). In the full sample, 49 percent of respondents met the threshold for a mental health disorder, including 36.8 percent of males and 61.2 percent of females. This rate was highest in the governorate of Qalqiya (60.42 percent) and lowest in the governorate of Jericho (36.11 percent).

Regarding risk-taking, 40.2 percent of respondents reported engaging in at least one risk behavior, including 57.7 percent of males and 22.6 percent of females. The most common risk behavior by far was smoking, which is far less stigmatized than drug use, drinking, and sexual intercourse outside of marriage. Only 10.7 percent of the sample reported engaging in a risk behavior other than smoking, with a significant difference by gender: the prevalence of engaging in one or more risk behaviors other than smoking was 15.5 percent among male youth and 4.9 percent among female youth.

### 3.2. Associations between proximity to infrastructure and mental health

In the unadjusted model estimates, proximity to checkpoints manned by Israeli soldiers was associated with increased risk of adverse mental health outcomes, with youth further from these checkpoints reporting fewer symptoms of mental distress (b = −0.05, 95% CI −0.084 to −0.015), as shown in Table 2. To provide a sense of the overall effect of the presence of checkpoints, an interdecile increase in distance from a checkpoint (i.e., moving from a cluster that is 1.3 km from the nearest checkpoint to a cluster that is 8.1 km from the nearest checkpoint) was associated with a 0.09 point decrease in the HSCL-25 self-reported measure of anxiety and depression symptomology, or a 5.1% relative decrease compared with the sample mean.

The adjusted estimates add individual exposure to trauma and other controls (estimates for all covariates are presented in Appendix Table A3). The statistically significant association between mental health and proximity to manned checkpoints becomes even larger after adjusting for individual exposure to conflict-related trauma and individual demographic and socioeconomic characteristics (Table 2). In the adjusted model, an interdecile increase in distance from a manned checkpoint was associated with a 0.11 point decrease in the HSCL-25 self-reported measure of anxiety and depression (b = −0.06, 95% CI −0.095 to −0.024), or a 6.1% relative decrease compared with the sample mean, indicating improved mental health as distance increased.

The correlation between proximity to checkpoints and poor mental health is slightly stronger among female youth. In unadjusted gender-specified model estimates, the negative correlation between distance from the nearest checkpoint and HSCL-25 scores is significant in the female-specific unadjusted model (b = −0.056, 95% CI −0.107 to −0.005), but not in the male-specific unadjusted model (b = −0.043, 95% CI −0.091 to 0.004). After adjusting for other covariates, the male-specific model estimates (b = −0.058, 95% CI −0.106 to −0.01) and female-specific model estimates (b = −0.061, 95% CI −0.111 to −0.011) are both significant, showing lower HSCL-25 scores among both male and female youth as distance from checkpoints increases, but the effect size remains slightly larger in the female subsample.

Comparing the effect sizes of proximity to checkpoints to those of direct and vicarious trauma exposure underscores the importance of checkpoint proximity. Like Wagner and colleagues (2020), who also analyze data from the Palestinian Youth Health Risk Survey, we find a significant association between both types of trauma and mental health. In the adjusted full-sample model for proximity to checkpoints, experience of direct trauma (b = 0.045, 95% CI 0.017 to 0.072) and vicarious trauma (b = 0.077, 95% CI 0.053 to 0.101) are both significantly and positively correlated with depression and anxiety symptoms (Appendix Table A3). An interdecile reduction in direct conflict-related trauma (i.e., a reduction from having experienced exactly one form of direct trauma to having experienced no direct trauma) was associated with a 0.04 point decrease in the HSCL-25, which represents a 2.5% decrease relative to the sample mean, and an interdecile reduction in vicarious conflict-related trauma (i.e., a reduction from two forms of vicarious trauma to no vicarious trauma) was associated with a 0.15 point decrease in the HSCL-25, which is an 8.5% decrease relative to the sample mean. As stated above, an interdecile change in distance to the nearest checkpoint is correlated with a 6.1% change in the HSCL-25 relative to the sample mean. The mental health effect of moving from one end to the other in the spatial distribution of checkpoint proximity is thus comparable to the effect of a similar movement in the distribution of trauma exposure. Furthermore, the effect of proximity to checkpoints on mental health is distinct from conflict-related trauma exposure, as the findings actually strengthen when controlling for trauma.

Like checkpoints, proximity to the other three types of occupation infrastructure – road obstructions, the separation barrier, and Israeli settlements – is positively associated with symptoms of poor mental health. However, these were not statistically significant in the full sample. In the gender-specified models, though, proximity to settlements was significantly correlated with poorer global mental health among female respondents (b = −0.048, 95% CI −0.096 to −0.001), as shown in Appendix Table A4. These significant associations appeared in both unadjusted and adjusted models. In the adjusted model estimates for female youth, an interdecile increase in distance from Israeli settlements was associated with a 0.09 point decrease in the HSCL-25, or a 5.1% relative decrease compared with the sample mean, indicating better mental health as distance increased.

### 3.3. Associations of proximity to infrastructure and risk behaviors

Several risk behaviors are significantly negatively associated with distance to one or more types of infrastructure in the adjusted estimates for the full sample (Table 3). The likelihood of reporting engagement in pre-marital sexual intercourse falls with distance from checkpoints (b = −0.022, 95% CI −0.037 to −0.007), road obstructions (b = −0.022, 95% CI −0.038 to −0.01), and the separation barrier (b = −0.009, 95% CI −0.017 to −0.001). The likelihoods of alcohol use (b = −0.016, 95% CI −0.030 to −0.001) and smoking also decrease with distance from checkpoints (b = −0.047, 95% CI −0.088 to −0.006). In contrast to these findings, the likelihood of illicit drug use increases with distance to most infrastructure types: checkpoints (b = 0.012, 95% CI 0.001 to 0.023), road obstructions (b = 0.010, 95% CI 0.000 to 0.020), and settlements (b = 0.014, 95% CI 0.004 to 0.024).

Disaggregating by gender reveals that the associations between risk behaviors and proximity to Israeli infrastructure generally exhibit different patterns for male and female youth, as shown in Table 4. The exception is the positive correlation between proximity to road obstructions and the likelihood of having had sexual intercourse; for both males and females, an interdecile increase in distance to the nearest road obstruction is associated with a 5-percentage point decrease in this probability. Other correlations, however, differ by gender. For example, for female youth, distance from checkpoints was strongly associated with a lower likelihood of having had sexual intercourse (among respondents aged 18 and over), drinking alcohol, and smoking, with no association with illicit drug use. In contrast, among male youth, no such significant negative correlations between distance from manned checkpoints and the first three risk behaviors were found, while there was a *positive* correlation with ever using illicit drugs (b = 0.033, 95% CI 0.013 to 0.053). This positive association was replicated in models for other infrastructure types. As shown in Table 4, the probability of illicit drug use among male youth significantly increases with distance from road obstructions (b = 0.027, 95% CI 0.009 to 0.044) and settlements (b = 0.028, 95% CI 0.009 to 0.048) as well. The correlation between male youth ever using illicit drugs and distance from the separation barrier is also positive, but not significant.

Distance from the separation barrier is similarly linked to divergent outcomes between female and male youth. While greater distance from the separation barrier is correlated with a lower likelihood of sexual intercourse among male youth (b = −0.013, 95% CI −0.023 to −0.002), it is correlated with a higher likelihood of smoking among female youth (b = 0.028, 95% CI 0.004 to 0.052).

### 3.4. Associations between individual-level characteristics, global mental health, and risk behaviors

A number of individual characteristics in the adjusted proximity models appear to be protective of youth mental health (though causality may run from better mental health to these factors, or the relationship may reflect unmeasured factors associated both with characteristics and outcomes). For males, being married, employed, and in school are associated with reduced HSCL-25 scores, indicating better mental health, across all four adjusted proximity models. In contrast, these factors do not show correlations with mental health for female youth. Instead, for female youth, there is a strong correlation across the four proximity models (b = −0.16, 95% CI −0.28 to −0.05) between having one’s father still living and reduced HSCL-25 scores.

With regard to impacts on risk behaviors, direct or vicarious experience of trauma (which, as already discussed, is associated with poorer mental health) appears to increase participation in some risk behaviors. Direct trauma experience is positively associated with smoking, alcohol use, and sexual intercourse among male youth, and positively associated with illicit drug use among both male and female youth. Vicarious trauma experience is associated with tobacco use for both males and females and with sexual intercourse for females.

In addition to trauma exposure, several individual-level characteristics—including those linked to age, setting, assets, employment, and education—are significantly correlated with risk behaviors. In all gender-specified adjusted proximity models, older age is associated with greater likelihood of engaging in all four risk behaviors among males, and ever drinking alcohol among females. The residential setting is also significant for risk behaviors. For males, living in an urban area (relative to rural, the base category) increased the likelihood of ever drinking and current smoking. For females, living in a refugee camp (relative to a rural setting) increased the likelihood of ever using illicit drugs. Greater household assets are associated with greater likelihood of multiple risk behaviors as well. Among male youth, household assets are positively correlated with the likelihood of drinking, smoking, and illicit drug use, while among female youth, household assets are positively correlated with the likelihood of drinking, smoking, and sexual intercourse. Additionally, employment among female youth is correlated with greater likelihood of ever drinking and smoking. Finally, and in contrast to the previously discussed characteristics, education is associated with lower risk-taking. For males, school enrollment is negatively correlated with the likelihood of smoking, and for females, years of education is negatively correlated with ever drinking alcohol and having had pre-marital sexual intercourse.

### 3.5. Sensitivity analyses

The sensitivity analyses confirmed the robustness of our findings, as alternative functional forms for measuring proximity did not substantively change the findings. Appendix Table A5 shows regression estimates for global mental health when employing alternate operationalizations of proximity to manned checkpoints.

## 4. Discussion

### 4.1. Summary and directions for future research

In this study we examined the association of proximity to the physical infrastructure of Israeli occupation in the West Bank with Palestinian youths’ mental health and risk-taking behavior. In regard to mental health outcomes, proximity to manned checkpoints was found to be significantly and consistently correlated with poorer mental health among both male and female youth, and proximity to settlements was associated with poorer mental health among female youth. Our findings suggest that the link between adverse mental health outcomes and proximity to manned checkpoints is not driven by individuals’ experiences of violence at checkpoints, nor is it reflective of a simple correlation between checkpoint proximity and individual experience of conflict-related violence. The observed relationship between proximity to manned checkpoints and poorer mental health did not attenuate, and in fact strengthened, after controlling for self-reported direct and vicarious trauma exposure. Similarly, the correlation between settlement proximity and poorer mental health among females was robust to controlling for conflict-related trauma exposure. These results point to the relevance of ecological models of mental health for populations living in conflict settings, indicating that chronic environmental factors play a significant role in mental health separately from conflict exposure.

While we show that the mental health effects of living near checkpoints (for all youth) and settlements (for female youth) are not mediated by conflict-related trauma, we can only propose hypotheses about what alternative mechanisms may be underlying these phenomena. Based on patterns in our results, we suggest that future research test whether the operative mechanism is increased (anticipation of) interaction with Israeli individuals associated with the occupation. We suggest this because our results show that proximity to infrastructure that entails the presence of Israeli individuals (i.e., manned checkpoints and settlements) is significantly correlated with poorer mental health, whereas proximity to unmanned infrastructure (i.e., road obstructions and the separation barrier) is not. While all of these infrastructure types restrict free movement, only those types that might occasion Israeli-Palestinian interpersonal contact are linked to mental health. Considering that exposure to violence does not mediate this association, we speculate that there may be non-violent aspects of interactions with Israelis in the West Bank that are nonetheless deleterious for the mental health of Palestinian youth. Prior research suggests these may include exposure to verbal abuse and humiliation (Barber et al., 2016; Giacaman et al., 2007), invasions of privacy (Sousa et al., 2014), and/or anticipatory stress regarding encounters with Israelis (Alang et al., 2021; Giacaman et al., 2009; Sousa et al., 2014). Further research is needed to test these explanations.

In regard to impacts on risk-taking, our analysis produced a more complex pattern of findings, with some notable differences by gender. For example, increased distance from manned checkpoints was associated with a decreased probability of female youth engaging in several risk behaviors, including pre-marital sexual intercourse, drinking alcohol, and smoking, but an increased probability of male youth engaging in illicit drug use. Furthermore, increased distance from the separation barrier was associated with a decrease in the probability of pre-marital sexual intercourse among male youth, but an increase in smoking among female youth. These findings thus suggest that proximity to certain types of occupation infrastructure impact young women’s and men’s engagement in risk behaviors differently.

Occupation infrastructure also may also affect certain risk behaviors differently than others, explaining some apparent puzzles in the findings. For example, while male youths’ likelihood of engaging in pre-marital sexual intercourse decreased with distance to multiple types of infrastructure, their likelihood of illicit drug use significantly increased with distance to checkpoints, road obstructions, and Israeli settlements. The inconsistency in the direction of associations across male risk-taking behaviors may reflect that the presence of occupation infrastructure has differential impacts on illegal activities compared to legal behaviors. For example, the heightened security associated with manned checkpoints and settlements could restrict the flow of illicit substances into nearby West Bank communities, or even directly inhibit drug use. Further research is needed to determine why the associations between proximity to occupation infrastructure and illicit drug use trend in the opposite direction from most other legal (if socially discouraged) risk behaviors.

### 4.2. Limitations

Despite the plausibility and robustness of the main findings for mental health, there remain challenges to a causal interpretation of these results, which arise mainly from the observational, cross-sectional nature of the data. Collinearity between different types of conflict-related infrastructure poses one challenge. For example, in additional analyses we found that proximity specifically to *concrete* segments of the separation barrier was significantly correlated with poorer mental health. However, the locations of concrete segments of the separation barrier are correlated with the locations of manned checkpoints: controlling for proximity to manned checkpoints eliminated the significance of proximity to the concrete segments of the barrier for mental health. While this result provides additional evidence for the significance of proximity to manned checkpoints for youth mental health, it also illustrates how the colocation of different types of occupation infrastructure can pose challenges to isolating specific effects.

Another and perhaps more significant challenge to causal interpretation comes from the potential correlation between proximity to occupation infrastructure and unmeasured locality characteristics that also affect mental health and other outcomes, which would lead to biased estimates of proximity effects. Estimations using governorate fixed effects, which relies solely on within-governate variation in regressors and outcomes, in principle would control for any unmeasured characteristics associated with governorate setting. However, the approach would also eliminate most of the sample variation in the distance measures, and indeed the estimates of the effects of distance to checkpoints were statistically insignificant when controls for governorate location were added. Hence it is not possible with our data to fully distinguish the impacts of proximity to infrastructure from other underlying governorate- or local-level factors that may correlate with both checkpoint placement and mental health, such as the intensity of conflict in a local area. Future research could generate more definitive causal inferences by collecting time-series or panel data to generate intra-governorate variation over time.

Lastly, our use of data from 2014 may raise concerns about the relevance of our findings to the present-day. If anything, their relevance has—unfortunately—increased. Compared to 2014, the number of checkpoints in the West Bank has more than doubled; nearly a hundred new settlements have been established; the number of road obstructions has increased; and part of the separation barrier has been converted from fencing to concrete (Fabian, 2022; Peace Now, n.d.; United Nations Office for the Coordination of Humanitarian Affairs - oPt, 2023a; 2023b). The expansion of occupation infrastructure reflects a trend that has been ongoing since 2014, but which accelerated after the start of the Israel-Hamas conflict in October 2023. Due to these changes in the landscape, it is likely that the factors examined in this study impinge even more strongly on Palestinian youth in the West Bank today. Additionally, in the post-October 2023 context, there has been a significant rise in settler violence and home demolitions in the West Bank (United Nations Office for the Coordination of Humanitarian Affairs -oPt, 2023c), suggesting that exposure to conflict-related trauma may have increased as well. These changes over time may affect the currentness of some of our descriptive summary statistics, but should not affect the validity of our analytical findings regarding relationships between occupation infrastructure and mental and behavioral health outcomes.

### 4.3. Conclusion

This study has demonstrated the usefulness of spatial data and methods to understanding the impacts of conflict—and of the occupation of the West Bank in particular—on youth mental health and behavior. The approach contributes to a more layered understanding of how conflict affects youth mental health. We showed that built features of the conflict and their spatial arrangement on the landscape are connected to the mental health and health behaviors of Palestinian youth living in the West Bank. Our results thus provide support for ecological models of mental health in conflict settings that look beyond direct experiences of war, as proximity to checkpoints was correlated with mental health independently of exposure to conflict-related violence.

This study has policy implications for ameliorating conflict-related threats to the mental health of Palestinian youth. While Palestinian authorities and international organizations currently have little to no say over the location and characteristics of Israeli infrastructure in the West Bank, the results can inform the targeting of mental health interventions across the area. Our findings suggest that as the physical infrastructure of the political conflict changes over time, the prevalence and geographic distribution of anxiety and depression may be changing alongside it. Understanding this spatial relationship could allow service providers and public health officials in the West Bank to efficiently allocate mental healthcare resources in response to changing local contexts and needs.

## Supplementary Material

1

## Figures and Tables

**Table 1:** Descriptive summary statistics.

	Full sample	Females	Males
**Mean age (SD) [Range]**	19 (2.84) [15–24]	19.14 (2.85) [15–24]	18.85 (2.82) [15–24]
**Years of education**			
8 or fewer	4.93%	3.68%	6.19%
12 or more (among those 18 years or older)	70.66%	74.18%	66.87%
No response	1.57%	1.89%	1.26%
**Enrolled in school**	63.44%	65.35%	61.52%
**Employed**	17.00%	4.85%	29.15%
No response	0.04%	0.09%	0%
**Ever married**	12.65%	22.80%	2.51%
**Residential Setting**			
Rural	29.43%	29.44%	29.42%
Urban	63.03%	63.02%	63.05%
Refugee camp	7.54%	7.54%	7.53%
**Mean asset index (SD) [Range]**	−0.04 (0.83) [−2.75–1.6]	−0.15 (0.84) [−2.75–1.6]	0.07 (0.81) [−2.61–1.6]
**Father experiencing hardship**	11.22%	11.76%	10.67%
No response	0.45%	0.63%	0.27%
**Father deceased**	5.56%	5.83%	5.29%
**Mother deceased**	1.12%	1.44%	0.81%
**Direct trauma exposure**	21.80%	7.18%	36.41%
No response	0.09%	0%	0.18%
**Vicarious trauma exposure**	73.53%	65.62%	81.43%
No response	0.13%	0%	0.27%
**Mean HSCL-25 score (SD) [Range]**	1.8 (0.47) [1–3.88]	1.93 (0.5) [1–3.88]	1.67 (0.4) [1–3.75]
**Mean depression score (SD) [Range]**	1.89 (0.51) [1–4]	2.02 (0.53) [1–4]	1.76 (0.44) [1–3.86]
**Mean anxiety score (SD) [Range]**	1.67 (0.51) [1–3.9]	1.80 (0.55) [1–3.9]	1.55 (0.43) [1–3.6]
**Ever drink alcohol**	7.81%	3.41%	12.20%
**Ever use illicit drugs**	3.32%	1.62%	5.02%
**Current smoker**	38.13%	21.01%	55.25%
**Sexual intercourse before marriage (asked to respondents aged 18 and older only)**	4.82% (excluding missing values)	4.41% (excluding missing values)	5.22% (excluding missing values)
**Total observations**	2229	1114	1115

**Table 2:** Association between global mental health and distance to the physical infrastructure of the Israeli occupation (OLS models).

	Manned checkpoints		Road obstructions		Separation barrier		Israeli settlement	
	Unadjusted	Adjusted	Unadjusted	Adjusted	Unadjusted	Adjusted	Unadjusted	Adjusted
Global mental health	−0.050** (−0.084 to −0.015) p = 0.0049	−0.060** (−0.095 to −0.024) p = 0.0011	−0.016 (−0.046 to 0.013) p = 0.28	−0.018 (−0.046 to 0.010) p = 0.20	−0.016 (−0.035 to 0.004) p = 0.11	−0.010 (−0.029 to 0.009) p = 0.29	−0.019 (−0.052 to 0.014) p = 0.25	−0.020 (−0.051 to 0.011) p = 0.20
Change associated with inter decile increase in distance	−0.091	−0.11	−0.038	−0.04	−0.050	−0.03	−0.037	−0.04
Change as percentage of mean (%)	−5.1%	−6.1%	−2.1%	−2.3%	−2.8%	−1.8%	−2.0%	−2.2%

Note: Each model only includes a single measure of distance, which is the natural logarithm of the distance in kilometers to the closest instance of the physical infrastructure type indicated in the column heading. All models were estimated using ordinary least squares with robust clustered SEs at the survey cluster level. Adjusted model results include conflict-related trauma (direct and indirect), individual demographics (age, gender, marital status, location of residence, parents deceased), and socioeconomic characteristics (household income, years of education, school enrollment, employment, and father experiencing hardship). In unadjusted models, *N* = 2228. In adjusted models, after dropping observations with missing data for covariates, *N* = 2149. 95% confidence intervals are shown in parentheses.

*** - p < 0.001,

** - p < 0.01,

* - p < 0.05.

**Table 3:** Associations between risk behaviors and distance to the physical infrastructure in adjusted probit conditional marginal effects for full sample.

	Manned checkpoints	Road obstructions	Separation barrier	Israeli settlements	*N*
Sexual intercourse	−0.022** (−0.04 to −0.01) p = 0.004	−0.022** (−0.04 to −0.01) p = 0.004	−0.009* (−0.02 to −0.00) p = 0.037	−0.007 (−0.02 to 0.01) p = 0.377	1201
Ever drink	−0.016* (−0.03 to −0.00) p = 0.034	−0.000 (−0.01 to 0.01) p = 0.987	−0.003 (−0.01 to 0.00) p = 0.413	0.004 (−0.01 to 0.02) p = 0.610	2144
Ever drugs	0.012* (0.00–0.02) p = 0.034	0.010* (0.00–0.02) p = 0.046	0.001 (−0.01 to 0.01) p = 0.854	0.014** (0.00, 0.02) p = 0.008	2119
Smoker	−0.047* (−0.09 to −0.01) p = 0.024	−0.022 (−0.06 to 0.01) p = 0.205	0.028** (0.01–0.05) p = 0.005	0.006 (−0.03 to 0.04) p = 0.739	2147

Note: Each model only includes a single measure of distance, which is the natural logarithm of the distance in kilometers to the closest instance of the physical infrastructure type indicated in the column heading, and models for each outcome measure were estimated separately. Models were estimated with a probit regression with robust clustered SEs at the survey cluster level. Each estimate presented is the marginal effect assessed at the mean of all covariates. All models control for exposure to conflict-related trauma, demographic characteristics, and socioeconomic characteristics. 95% confidence intervals are shown in parentheses.

*** - p < 0.001,

** - p < 0.01,

* - p < 0.05.

**Table 4:** Associations between risk behaviors and distance to the physical infrastructure in adjusted probit models with conditional marginal effects for female and male subsamples.

	Manned checkpoints	Road obstructions	Separation barrier	Israeli settlements	*N*
**Females**					
Sexual intercourse	−0.024** (−0.04 to −0.01) p = 0.007	−0.022* (−0.04 to −0.00) p = 0.011	−0.006 (−0.02 to 0.00) p = 0.215	−0.003 (−0.02 to 0.02) p = 0.718	589
Ever drink	−0.015** (−0.03 to −0.00) p = 0.008	−0.007 (−0.02 to 0.00) p = 0.183	−0.003 (−0.01 to 0.00) p = 0.373	−0.002 (−0.01 to 0.01) p = 0.745	940
Ever drugs	−0.005 (−0.01 to 0.00) p = 0.133	−0.005 (−0.01 to 0.00) p = 0.209	−0.001 (−0.01 to 0.00) p = 0.491	0.000 (−0.01 to 0.01) p = 0.972	1047
Smoker	−0.070** (−0.12 to −0.02) p = 0.004	−0.036 (−0.08 to 0.01) p = 0.101	0.028* (0.00–0.05) p = 0.023	−0.012 (−0.05 to 0.03) p = 0.534	1067
**Males**					
Sexual intercourse	−0.018 (−0.04 to 0.00) p = 0.070	−0.022* (−0.04 to −0.00) p = 0.042	−0.013* (−0.02 to −0.00) p = 0.015	−0.009 (−0.03 to 0.01) p = 0.404	604
Ever drink	−0.018 (−0.05 to 0.01) p = 0.201	0.009 (−0.02 to 0.03) p = 0.471	−0.004 (−0.02 to 0.01) p = 0.557	0.012 (−0.02 to 0.04) p = 0.429	1078
Ever drugs	0.033** (0.01–0.05) p = 0.001	0.027** (0.01–0.04) p = 0.002	0.005 (−0.01 to 0.00) p = 0.367	0.028** (0.01–0.05) p = 0.004	1072
Smoker	−0.002 (−0.05 to 0.05) p = 0.947	−0.005 (−0.05 to 0.04) p = 0.824	0.016 (−0.01 to 0.04) p = 0.215	0.024 (−0.02 to 0.07) p = 0.300	1080

Note: Each model only includes a single measure of distance, which is the natural logarithm of the distance in kilometers to the closest instance of the physical infrastructure type indicated in the column heading, and models for each outcome measure were estimated separately. Models were estimated with a probit regression with robust clustered SEs at the survey cluster level. Each estimate presented is the marginal effect assessed at the mean of all covariates. All models control for exposure to conflict-related trauma, demographic characteristics, and socioeconomic characteristics. 95% confidence intervals are shown in parentheses.

*** - p < 0.001,

** - p < 0.01,

* - p < 0.05.

## Data Availability

The data from the Palestine Youth Health Risk Survey cannot be publicly shared due to confidentiality concerns. Data on manned checkpoints and unmanned road obstructions were obtained from the Applied Research Institute of Jerusalem (ARIJ). The authors are not at liberty to share ARIJ’s data. Data on Israeli settlements are publicly available and can be downloaded from B’Tselem at this URL: https://www.btselem.org/download/settlement_population.xls. Data on the separation barrier are publicly available and can be viewed or downloaded from the UN Office for the Coordination of Humanitarian Affairs at this URL: https://data.humdata.org/dataset/west-bank-barrier.

## Footnotes

1 While some settlements are legal under Israeli law, Israeli settlements in the West Bank are considered illegal under international law, namely the Fourth Geneva Convention and the Hague Regulations (International Court of Justice, 2024; Lein and Weizman, 2002).

2 Additional clusters located in East Jerusalem were not included in this analysis both because of high non-response to the survey (30% vs. 8% elsewhere on average) and because political and administrative factors affecting mobility and mobility-restricting infrastructure differ significantly between densely populated East Jerusalem and the West Bank.

3 After dropping observations with missing data on outcomes, our data include 183 survey clusters with 12 respondents each and 4 survey clusters with 8 or 9 respondents each.

## Appendix A. Supplementary data

Supplementary data to this article can be found online at https://doi.org/10.1016/j.healthplace.2025.103420.
