# Supplementary material for: Assessing the mental health impacts of Israeli occupation infrastructure in the West Bank by combining geospatial data with a representative survey of Palestinian youth
Source: Health Place. Author manuscript; Available in PMC 2026 Jun 9. (PMC13248938; doi:10.1016/j.healthplace.2025.103420)
Supplement: 1 [file NIHMS2170773-supplement-1.pdf]

Assessing the Mental Health Impacts of Israeli Occupation Infrastructure in the West Bank by Combining Geospatial Data with a Representative Survey of Palestinian Youth

Appendix

Section A1. Geospatial Data

Our analysis identifies four main types of physical infrastructure: the separation barrier, Israeli settlements, checkpoints, and road obstructions. The separation barrier includes concrete stretches of wall, fencing, and wide land barrier with barbed-wire electric fences on both sides. Settlements include Israeli settlements and outposts, both those considered illegal and legal under Israeli law. Checkpoints are mobility controls staffed by Israeli security agents, whether located along the separation barrier or internally within the West Bank. Partial checkpoints, which are checkpoints that are only sometimes staffed, are included under this type of infrastructure. Road obstructions are unmanned mobility controls, including road gates, roadblocks, and earth mounds. The dataset included 104 road gates, which are metal gates controlling road access. The data also included 62 roadblocks, which are road obstructions made of concrete blocks. Earth mounds are mounds of dirt, rubble, and/or rocks laid across roads to block traffic, and the data captured 144 earth mounds.

Data on road obstructions and manned checkpoints throughout the West Bank and East Jerusalem were supplied by the Applied Research Institute of Jerusalem (ARIJ). The ARIJ dataset identified the infrastructure by subtype and provided their location coordinates. The dataset includes time-series data showing the historical presence of each piece of infrastructure, but this study analyzes only the infrastructure present in the year 2014. Data on settlement locations in 2014 came from a public dataset produced by the Israeli human rights organization B’Tselem (2019). Data on the location and subtypes of the separation barrier came from a public dataset produced by the United Nations Office for Coordination of Humanitarian Affairs (2018).

Geospatial data on the infrastructure in place in 2014 was merged with the Palestinian Youth Health Risk Survey, which was collected in 2014 (see below). The survey data included the location coordinates of the centroid of respondents’ survey clusters. By relating survey cluster centroid coordinates to infrastructure coordinates, we were able to calculate survey respondents’ distance—at the cluster level—to various forms of infrastructure, using multiple functional forms of the distance measure to check for robustness.

The analysis in the main paper and in tables A3 and A4 use the logarithm of the Euclidean distance between the survey cluster centroid and the nearest checkpoint, road obstruction, settlement, or segment of the separation barrier. The Euclidean distance denotes the shortest straight-line distance between two points. The sensitivity analysis, presented in table A5, applies other functional forms of the distance measure, such as the Euclidean distance to the nearest checkpoint; the inverse of distance to the nearest checkpoint (e.g., ¼ , where 4 is the distance in kilometers to the nearest checkpoint), in negative form; binary indicators for whether the nearest checkpoint is within 1 kilometer or 2 kilometers, in negative form; the sum of inverse distances to all pieces of infrastructure within a 5-km, 10-km, and 20-km radius, in negative form; and the quantity of infrastructure pieces within a 5-km, 10-km, and 20-km radius, in negative form.

Negative forms are used for some of these measures so that a larger value for the measure indicates reduced proximity to infrastructure, consistent with the direction of the estimates for the baseline specification (i.e., logarithm of distance).

Section A2. Survey Data

The Palestinian Youth Health Risk Survey has been used in other published papers that assess Palestinian youth mental health and health risk behaviors (Glick et al., 2018b, 2018a; Wagner et al., 2020). Survey data collection procedures were approved by RAND Corporation Human Subjects Protection Committee. The survey was conducted between April and July 2014 and includes 2,500 youth (ages 15-24) in the West Bank and East Jerusalem, selected based on a stratified two-stage random sample. The first stage of the stratified random sample was designed to sample urban, rural, and refugee camp locations within each of the 12 governorates. From within these locations, 208 census enumerations areas were randomly sampled to form the survey clusters, with the probability of random selection proportional to enumeration areas' population sizes in the 2007 census. Within each cluster, 12 households with youth were chosen via modified random walk, using implicit stratification to ensure equal numbers by gender. In each household, youth were selected and interviewed. In households where there was more than one youth between 15-24 years old, one of the youths was selected at random and interviewed.

Interviews were conducted face to face, but some non-minors (older than 18) were chosen at random to fill out the sexual activity portion of the survey using a self-administered questionnaire. Surveys were conducted in a private location, such as a private room or the roof of the house. Participants were offered the option of conducting the survey at an outside location, like a local youth center, but few selected this option. Verbal consent was obtained from all youth over 18 years old, and parental verbal consent was obtained to interview minors. The overall refusal rate was 11%; the refusal rate in East Jerusalem was approximately 30%. The refusal rate in the rest of the West Bank was about 8%. Survey responses from East Jerusalem have been excluded from the analysis in this paper.

The survey covers questions on mental health, engagement in behaviors affecting health, risk perceptions and optimism for the future. For each survey cluster, the survey team measured the latitude and longitude of the approximate centroid of the 12 sampled households. This geographic data allowed us to link the survey data at the cluster level to the coordinates relating to the physical manifestations of the Israeli presence. Additionally, the analysis in this paper only includes clusters that are within the West Bank (187 of 208 clusters), leaving the youth who reside in East Jerusalem out of the analysis based on the assumption that these youth live under different conditions. However, infrastructure within East Jerusalem remains in the geospatial data, since for nearby clusters distance to infrastructure located in East Jerusalem remains relevant. For further details on the youth survey methods, see Glick et al (2018b).

A limitation of our study is that it examines data from a single time-point in youths' lives. The analysis links spatial data on respondents' place of residence and infrastructure as observed in 2014. Our analysis does not account for how proximity to infrastructure may have changed over a respondent's lifetime, for example due to the respondent's family relocating or due to changes in the spatial distribution of infrastructure.

Another limitation is that in an effort to protect respondents' confidentiality, respondents' home locations were not recorded. Instead, the research administering the survey recorded the approximate location of the centroid of each 12-household cluster. We do not know the exact radius of these clusters. Therefore, while distance from a piece of physical infrastructure to a cluster centroid could be 1 kilometer, the distance to a respondent's actual place of residence could be 0.75 kilometers or 1.25 kilometers, for example. Generally however, given the small area covered by individual clusters, which interviewers traversed on foot, this is not likely to be a serious issue.

Appendix References

B'Tselem, 2019. Statistics on Settlements and Settler Population. Available at: https://www.btselem.org/download/settlement_population.xls.

Glick, P., Al-Khammash, U., Shaheen, M., Brown, R., Goutam, P., Karam, R., Linnemayr, S., Massad, S., 2018a. Health risk behaviours of Palestinian youth: findings from a representative survey. East. Mediterr. Health J. 24, 127–136.

Glick, P., Khammash, U., Shaheen, M., Brown, R., Goutam, P., Karam, R., Linnemayr, S., Massad, S., 2018b. Perceived peer norms, health risk behaviors, and clustering of risk behaviors among Palestinian youth. PLOS ONE 13, e0198435. https://doi.org/10.1371/journal.pone.0198435.

Office for the Coordination of Humanitarian Affairs oPt, 2018. West Bank Separation Barrier. Humanit. Data Exch. https://data.humdata.org/dataset/west-bank-barrier. (Accessed 15 August 2022).

Wagner, G., Glick, P., Khammash, U., Shaheen, M., Brown, R., Goutam, P., Karam, R., Linnemayr, S., Massad, S., 2020. Exposure to violence and its relationship to mental health among young people in Palestine. East. Mediterr. Health J. 26, 189–197. https://doi.org/10.26719/2020.26.2.189.

Table A1 – Correlation Matrix among Outcome Variables

	Global mental health	Depression	Anxiety	Sexual intercourse	Ever drugs	Ever drink	Smoker
**Global mental health**	1***						
**Depression**	0.95***	1***					
**Anxiety**	0.90***	0.73***	1***				
**Sexual intercourse**	0.16***	0.17***	0.12***	1***			
**Ever drugs**	0.04	0.04	0.03	0.28***	1***		
**Ever drink**	0.11***	0.12***	0.08***	0.31***	0.26***	1***	
**Smoker**	0.05*	0.06**	0.04	0.19***	0.15***	0.29***	1***

Notes:
*** - p<0.001 ** - p<0.01, * - p<0.05

Table A2 – Deciles for Distances between Survey Clusters and Each Infrastructure Type

	Checkpoints	Road Obstructions	Separation Barrier	Settlements
**10th**	1.3 km	1.1 km	0.6 km	1.3 km
**20th**	1.8 km	1.5 km	1.2 km	1.7 km
**30th**	2.3 km	1.7 km	2.4 km	2.0 km
**40th**	2.7 km	2.1 km	3.7 km	2.4 km
**50th**	3.4 km	2.5 km	5.5 km	2.9 km
**60th**	4.1 km	2.9 km	7.8 km	3.5 km
**70th**	5.3 km	3.7 km	10.8 km	4.0 km
**80th**	6.2 km	4.7 km	12.9 km	4.7 km
**90th**	8.1 km	10.7 km	14.4 km	9.0 km

Table A3 – Full Adjusted Regression Results for Proximity to Checkpoints for Each Outcome

	Global Mental Health	Depression	Anxiety	Currently Smoking	Sexual Intercourse	Ever Drugs	Ever Drink
**Distance from checkpoints**	-0.06**	-0.07**	-0.05*	-0.05*	-0.02**	0.01*	-0.02*
	[-0.09, -0.02]	[-0.11, -0.03]	[-0.08, -0.01]	[-0.09, -0.01]	[-0.04, -0.01]	[0.00, 0.02]	[-0.03, -0.00]
**Conflict-related trauma**							
Direct	0.04**	0.05**	0.04**	0.05**	0.01	0.01**	0.03***
	[0.02, 0.07]	[0.02, 0.08]	[0.01, 0.07]	[0.02, 0.09]	[-0.00, 0.02]	[0.00, 0.02]	[0.02, 0.04]
Vicarious	0.08***	0.08***	0.08***	0.10***	0.01	0.00	0.00
	[0.05, 0.10]	[0.05, 0.10]	[0.05, 0.10]	[0.07, 0.13]	[-0.00, 0.02]	[-0.00, 0.01]	[-0.01, 0.01]
**Individual-Level Characteristics**							
Age	0.02*	0.02*	0.02*	0.03***	0.01*	0.00**	0.01***
	[0.00, 0.03]	[0.00, 0.03]	[0.00, 0.03]	[0.02, 0.04]	[0.00, 0.01]	[0.00, 0.01]	[0.01, 0.02]
Female Gender	0.31***	0.32***	0.30***	-0.28***	0.03*	-0.01*	-0.04***
	[0.26, 0.36]	[0.26, 0.37]	[0.25, 0.36]	[-0.34, -0.22]	[0.01, 0.06]	[-0.03, -0.00]	[-0.06, -0.02]
Ever Married	-0.09*	-0.11*	-0.06	-0.07	-0.01	-0.01	-0.04*
	[-0.16, -0.01]	[-0.19, -0.02]	[-0.14, 0.02]	[-0.15, 0.01]	[-0.05, 0.02]	[-0.04, 0.01]	[-0.07, -0.00]
Live in Camp (ref: Rural)	-0.11*	-0.10*	-0.12*	0.05	0.01	0.04**	0.013
	[-0.20, -0.01]	[-0.20, -0.01]	[-0.23, -0.01]	[-0.07, 0.16]	[-0.03, 0.05]	[0.01, 0.06]	[-0.02, 0.05]
Urban (ref: Rural)	-0.01	-0.01	-0.03	0.09**	0.02	0.02*	0.03*
	[-0.07, 0.04]	[-0.06, 0.05]	[-0.08, 0.03]	[0.03, 0.15]	[-0.01, 0.04]	[0.00, 0.04]	[0.00, 0.05]
Father Alive	-0.12**	-0.12**	-0.12*	-0.03	0	0	-0.00
	[-0.20, -0.04]	[-0.21, -0.04]	[-0.21, -0.02]	[-0.13, 0.07]	[-0.03, 0.03]	[-0.02, 0.03]	[-0.04, 0.03]
Mother Alive	-0.2	-0.26	-0.12	-0.09	0.01	.	-0.04
	[-0.44, 0.05]	[-0.53, 0.02]	[-0.35, 0.11]	[-0.31, 0.14]	[-0.06, 0.07]	[-0.06, 0.07]	[-0.10, 0.03]
Asset Index	0	0	0	0.07***	0.02**	0.01*	0.02***
	[-0.03, 0.03]	[-0.03, 0.03]	[-0.03, 0.03]	[0.04, 0.11]	[0.00, 0.03]	[0.00, 0.02]	[0.01, 0.04]
Years Education	-0.01	0	-0.01	-0.02*	-0.01*	0	-0.00
	[-0.02, 0.01]	[-0.02, 0.01]	[-0.02, 0.00]	[-0.03, -0.00]	[-0.01, -0.00]	[-0.00, 0.00]	[-0.01, 0.00]
Father Hardship	-0.02	-0.02	-0.02	-0.04	0	0	-0.01
	[-0.09, 0.05]	[-0.10, 0.06]	[-0.09, 0.05]	[-0.12, 0.03]	[-0.03, 0.03]	[-0.02, 0.02]	[-0.03, 0.02]
Employed	-0.09**	-0.11**	-0.06	0.06	0.02	-0.01	0.01
	[-0.15, -0.03]	[-0.18, -0.04]	[-0.13, 0.00]	[-0.01, 0.13]	[-0.00, 0.05]	[-0.02, 0.01]	[-0.01, 0.04]
In School	-0.06	-0.08*	-0.03	-0.13***	-0.01	0.01	-0.01
	[-0.13, 0.01]	[-0.16, -0.01]	[-0.10, 0.04]	[-0.20, -0.05]	[-0.03, 0.01]	[-0.01, 0.02]	[-0.03, 0.01]
Observations	2,149	2,149	2,149	2,147	1,201	2,119	2,144

Notes:

Models are identical to the full-sample adjusted estimates for manned checkpoints in Tables 2 and 3 of the main paper. Each column represents a separate regression, with the dependent variables noted in the column header. The

estimates for currently smoking, risky sex, drug use, and alcohol use were obtained from marginal effects probit models evaluated at the mean of the independent variables. All other coefficients were computed using Ordinary Least Squares. 95% confidence intervals, computed using standard errors corrected for clustering at the survey cluster, are in parentheses with p-values as follows: *** - $p<0.001$, ** - $p<0.01$, * - $p<0.05$.

Table A4 – Gender-Specified Adjusted Regression Results for Proximity to Infrastructure and Global Mental Health

	Checkpoints		Settlements	
	**Female**	**Male**	**Female**	**Male**
**Distance from infrastructure**	-0.061*	-0.058*	-0.048*	0.008
	[-0.111, -0.011]	[-0.106, -0.010]	[-0.096, -0.001]	[-0.031, 0.047]
**Conflict-related trauma**				
Direct	0.090	0.043**	0.084	0.044**
	[-0.033, 0.212]	[0.013, 0.073]	[-0.039, 0.207]	[0.013, 0.075]
Indirect	0.084**	0.067**	0.085**	0.066**
	[0.048, 0.120]	[0.036, 0.097]	[0.049, 0.121]	[0.035, 0.097]
**Individual-Level Characteristics**				
Age	0.018	0.015	0.017	0.013
	[-0.003, 0.039]	[-0.002, 0.032]	[-0.004, 0.038]	[-0.004, 0.030]
Ever Married	-0.072	-0.160*	-0.070	-0.145*
	[-0.165, 0.021]	[-0.297, -0.024]	[-0.163, 0.023]	[-0.279, -0.010]
Live in Camp (ref: Rural)	-0.125	-0.091	-0.103	-0.077
	[-0.273, 0.023]	[-0.222, 0.040]	[-0.257, 0.050]	[-0.199, 0.046]
Urban (ref: Rural)	0.001	-0.027	0.013	-0.020
	[-0.075, 0.077]	[-0.097, 0.043]	[-0.064, 0.090]	[-0.091, 0.050]
Father Alive	-0.166**	-0.065	-0.166**	-0.059
	[-0.280, -0.052]	[-0.185, 0.054]	[-0.282, -0.050]	[-0.179, 0.060]
Mother Alive	-0.126	-0.304	-0.132	-0.305
	[-0.376, 0.124]	[-0.804, 0.196]	[-0.397, 0.134]	[-0.812, 0.202]
Asset Index	-0.006	0.008	-0.003	0.012
	[-0.048, 0.035]	[-0.028, 0.043]	[-0.044, 0.038]	[-0.025, 0.049]
Years of Education	-0.014	-0.000	-0.012	-0.00
	[-0.036, 0.008]	[-0.019, 0.019]	[-0.034, 0.010]	[-0.02, 0.02]
Father Hardship	-0.022	-0.027	-0.015	-0.02
	[-0.120, 0.077]	[-0.122, 0.067]	[-0.115, 0.084]	[-0.12, 0.07]
Employed	-0.018	-0.104**	-0.011	-0.10**
	[-0.156, 0.120]	[-0.171, -0.036]	[-0.149, 0.127]	[-0.17, -0.03]
In School	-0.035	-0.086*	-0.031	-0.09*
	[-0.136, 0.067]	[-0.170, -0.001]	[-0.135, 0.072]	[-0.17, -0.00]
Observations	1,068	1,081	1,068	1,081

Notes:
Each column represents a separate regression where the dependent variable is global mental health. Negative coefficients indicate an association with lower scores on the symptoms checklist, meaning better mental health. Coefficients were computed using Ordinary Least Squares. 95% confidence intervals, computed using standard errors corrected for clustering at the survey cluster, are in parentheses with p-values as follows: *** - p<0.001, ** - p<0.01, * - p<0.05.

Table A5 – Estimates for Global Mental Health using Alternative Functional Form for Proximity to Checkpoints

	Estimate	95% CI	P-Value	Change associated with interdecile increase in distance (% of mean)
**Closest manned checkpoint**				
Natural logarithm of Euclidean distance	-0.060***	(-0.095, -0.024)	[0.001]	-6.1%
Euclidean distance	-0.015***	(-0.024, -0.007)	[0.000]	-5.8%
Inverse of distance†	-0.106*	(-0.193, -0.018)	[0.018]	-3.8%
Within 1-km (dichotomous)†	-0.035	(-0.130, 0.061)	[0.480]	--
Within 2-km (dichotomous)†	-0.060*	(-0.118, -0.002)	[0.044]	-3.3%
**Sum of manned checkpoints within radius, inverse weighted**				
5-km†	-0.054**	(-0.092, -0.017)	[0.005]	-5.4%
10-km†	-0.053***	(-0.080, -0.025)	[0.000]	-6.6%
20-km†	-0.052***	(-0.076, -0.029)	[0.000]	-8.0%
**Sum of manned checkpoints within radius, unweighted**				
5-km†	-0.025***	(-0.038, -0.013)	[0.000]	-7.1%
10-km†	-0.015***	(-0.021, -0.009)	[0.000]	-8.3%
20-km†	-0.009***	(-0.013, -0.005)	[0.000]	-8.9%

Notes:

Models are identical to the adjusted estimates in Column 1 of Table 2 of the main paper, except here we vary the specification of the functional form for the proximity to the manned checkpoints. The first row repeats the analysis presented in Column 1 of Table 2. †: Functional form is expressed in negative terms in the analysis so that a larger value for the measure indicates reduced proximity of checkpoints, in order to be consistent with the direction of the estimates for the baseline specification. Models were adjusted for conflict-related trauma (direct and indirect), individual demographics (age, gender, marital status, location of residence, parents deceased), and socioeconomic characteristics (household income, years of education, and school enrollment, employment, father financial hardship). The change in the outcome associated with increased proximity from the 10th to the 90th percentile varies by the specific functional form. The interdecile difference cannot be calculated for “Within 1-km” as less than 10 percent of observations are within 1-km. 95% confidence intervals, computed using standard errors corrected for clustering at the survey cluster, are in parentheses. Exact p-values are provided in square brackets.

*** - p<0.001, ** - p<0.01, * - p<0.05.
